# Evaluation of numerical schemes for capturing shock waves in modeling proppant transport in fractures

**DOI:** 10.1007/s12182-017-0194-x

**Published:** 2017-11-01

**Authors:** Morteza Roostaei, Alireza Nouri, Vahidoddin Fattahpour, Dave Chan

**Affiliations:** Department of Civil and Environmental Engineering, University of Alberta, Edmonton, Canada

**Keywords:** Proppant transport, Hyperbolic partial differential equations, Frac pack, Hydraulic fracturing

## Abstract

In petroleum engineering, the transport phenomenon of proppants in a fracture caused by hydraulic fracturing is captured by hyperbolic partial differential equations (PDEs). The solution of this kind of PDEs may encounter smooth transitions, or there can be large gradients of the field variables. The numerical challenge posed in a shock situation is that high-order finite difference schemes lead to significant oscillations in the vicinity of shocks despite that such schemes result in higher accuracy in smooth regions. On the other hand, first-order methods provide monotonic solution convergences near the shocks, while giving poorer accuracy in the smooth regions. Accurate numerical simulation of such systems is a challenging task using conventional numerical methods. In this paper, we investigate several shock-capturing schemes. The competency of each scheme was tested against one-dimensional benchmark problems as well as published numerical experiments. The numerical results have shown good performance of high-resolution finite volume methods in capturing shocks by resolving discontinuities while maintaining accuracy in the smooth regions. These methods along with Godunov splitting are applied to model proppant transport in fractures. It is concluded that the proposed scheme produces non-oscillatory and accurate results in obtaining a solution for proppant transport problems.

## Introduction

It is well known that the hyperbolic partial differential equations (PDEs) accept both smooth and discontinuous solutions. A discontinuous solution, also referred to as a shock, is characterized by large gradients in the variables such as velocity, density (concentration), depth or pressure. Even with smooth initial conditions, discontinuities may develop with time (Chen 2006). In this paper, various methods of solving hyperbolic equations are investigated with the purpose of applying the best shock-capturing scheme to the proppant transport problem. To achieve this, some of the available shock-capturing techniques were employed in solving benchmark test problems and comparing the results. From a mathematical point of view, proppant transport equations are time-dependent, nonlinear hyperbolic PDEs. These kinds of PDEs are based on conservation laws and have applications in many engineering problems (LeVeque 2004).

The primary focus of this work is the modeling of proppant transport by solving the advection equation. Therefore, it is necessary to present a literature review on previous numerical proppant transport modeling. In the simplest form of the proppant transport models, such as those proposed by Daneshy (1978) and Novotny (1977), a vertical fracture is assumed and discretized into slim vertical sections (columns). Treatment time is also divided into small time increments. At each time increment, the fluid loss, the increase in sand concentration, the sand settling velocity, the volume and height of the deposited sand, and the height of the sand in suspension are computed based on the mass balance of different phases. These models, which also use an experimental correlation for the settling velocity of proppants, are called simplified models of proppant transport. They do not numerically solve the hyperbolic partial differential equation of proppant transport and are limited to planar vertical fractures.

In the next generation of proppant transport models, mixture-type models were developed to overcome the restrictions of the simplified models. In the original model, several assumptions were made regarding the proppant condition inside the fracture. The proppant and carrying fluid velocities were assumed to be the same. In other words, it was assumed that no momentum transfer occurred between the carrying fluid and the granular phase. Also, no dispersion of proppant particles was considered; thus, the front of the proppant concentration profile remained sharp (Adachi et al. 2007). An essential element of these models is the averaging of the field variables, such as the particle volume fraction and the fluid velocity, in the direction perpendicular to the fracture walls. This assumption was the result of avoiding high computational cost of discretization across the width of the fracture. Although we did not try to resolve this issue, the method of dimensional splitting presented in this paper can be used for capturing the variations along the fracture width.

Many modifications have been made in the original mixture models by different researchers to improve the simulation techniques for the transport phenomena. Settari et al. (1990) were the first to propose the concept of partially decoupled fracture modeling. They linked a fracture simulator, a fluid flow simulator and a proppant simulator together and mapped the fracture geometry and proppant concentration in terms of permeability and porosity onto the reservoir simulator grids. To better capture the discontinuous front of proppants inside the fracture, Settari et al. (1990) used a finer finite difference grid (finer than the grids used in the fracture and fluid flow modules) for solving the one-dimensional PDE of the proppant transport model. Later, several researchers used this approach in their numerical simulation of proppant transport. Behr et al. (2006), Shaoul et al. (2007) and Miranda et al. (2010) linked a commercial reservoir simulator to a commercial fracture and proppant simulator and used the same concept that Settari et al. (1990) had used for frac pack analysis. Although the linking of proppant and fracture simulators was novel, the proppant transport in these works was not numerically modeled by solving the mass balance hyperbolic PDE.

Later other models have been proposed to simulate proppant transport numerically (Friehauf 2009; Gadde et al. 2004; Liu 2006; Ouyang 1994; Sharma and Gadde 2005). The main focus of these models was simulating transport in multi-phase and multi-component slurries. Ouyang (1994) proposed an adaptive meshing technique in the hydraulic fracture simulation that was combined with the fully decoupled models developed by Ribeiro (2013) for proppant injection simulations. Little attention was given to the efficiency of the numerical scheme in solving the proppant transport equations. Gadde et al. (2004), Liu and Sharama (2005), Liu (2006) and Friehauf (2009) used the Perkins–Kern–Nordgren (PKN) fracture geometry and included some of the experimental works in the literature related to proppant transport in their numerical model. The finite difference scheme was employed to solve the transport equation similarly with little attention to the efficiency of the numerical scheme used.

In this research, different shock-capturing schemes have been investigated for solving 1-D and 2-D proppant transport equations. For 2-D equations, the operator splitting technique is employed for the treatment of the source terms and the multi-dimensionality of the problem. The high-resolution method of finite volume is applied through the application of flux limiters to solve the transport equations. Although such schemes slightly increase the computational complexity, they can achieve comparable results with a coarser spatial resolution (LeVeque 2004). These schemes have demonstrated very promising shock-capturing capabilities since they can achieve good accuracy while avoid spurious oscillations.

## Theory and governing equations of slurry proppant transport

The process of transport of material can be captured by a system of hyperbolic partial differential equations. The general one-dimensional form of these conservation laws can be written as:

1 $$\frac{\partial q}{\partial t} + \frac{\partial f}{\partial x} = 0$$

where $t$ is time, $x$ is the horizontal coordinate, $q$ is the conserved variable to be advected (e.g., in proppant transport we will see that it is concentration multiplied by the width of the fracture) and $f$ is the flux vector. (In proppant transport, it is velocity multiplied by concentration multiplied by width of the fracture.) In many applications, the flux vector *f* can be defined as:

2 $$f = uq$$

where $u$ is the velocity of propagation. With this definition, the conservation form of the hyperbolic equation reduces to an advection form:

3 $$\frac{\partial q}{\partial t} + \frac{{\partial \left( {uq} \right)}}{\partial x} = 0$$

This form of advection equation in proppant transport problem is nonlinear since the velocity of proppant changes with space and time. Therefore, special techniques suitable for nonlinear hyperbolic problems must be utilized. However, if we assume that we are dealing with an incompressible fluid, a major simplification can be made. For incompressible fluids in 1-D, from continuity equation one can write:

4 $$u_{x} = 0$$

This equation is equivalent to the conservation of mass and states that the velocity (rate) of the material conserved is the same in each section. Then Eq. (3) can be expanded as:

5 $$\frac{\partial q}{\partial t} + u\frac{\partial q}{\partial x} + q\frac{\partial u}{\partial x} = 0$$

Applying the continuity equation, we obtain:

6 $$\frac{\partial q}{\partial t} + u\frac{\partial q}{\partial x} = 0$$

This form of the advection equation is linear and is relatively easier to deal with (LeVeque 2004). In solving this kind of hyperbolic PDEs, different capturing schemes have been developed which can be classified as classical or traditional and modern techniques.

In the traditional techniques, the finite difference method is employed, while in the modern techniques, the finite volume method (which for rectangular grids can be viewed as a generalization of the finite difference method) is used. Here, we briefly present the finite volume discretization of the hyperbolic equations presented above.

In the finite volume method, the integral form of the partial differential equations is developed. In one-dimensional space, the finite volume discretization of Eq. (1) is:

7 $$Q_{i}^{n + 1} = Q_{i}^{n} - \frac{\Delta t}{\Delta x}\left( {F_{{i + \frac{1}{2}}}^{n} - F_{{i - \frac{1}{2}}}^{n} } \right)$$

where *Q* and *F* which are defined in Eqs. (8) and (9) are numerical solution to the PDE and numerical flux, respectively. All the terms in the above discretization are average values of the variables over the *i*th interval and at time *t*_*n*_ or *t*_*n*+1_, e.g.,

8 $$Q_{i}^{n} \approx \frac{1}{\Delta x}\mathop \int \limits_{{x - \frac{i}{2}}}^{{i + \frac{1}{2}}} q\left( {x,t_{n} } \right){\text{d}}x$$

9 $$F_{{i - \frac{1}{2}}}^{n} \approx \frac{1}{\Delta t}\mathop \int \limits_{{t_{n} }}^{{t_{n + 1} }} f\left( {q\left( {x_{{i - \frac{1}{2}}} ,t} \right)} \right){\text{d}}t$$

Any numerical method for solving hyperbolic equations depends on the choice of $F$, which is called the numerical flux function.

Since the solution of this kind of PDE may involve shocks in the solution, shock-capturing methods with the ability of tracking discontinuities and maintaining accuracy and stability in smooth regions have been developed (LeVeque 2004). In the next section, a review of the conventional finite difference methods and recent shock-capturing methods is provided. Our approach should not be confused with shock tracking or front tracking methods in which a combination of the finite difference or finite volume methods (in smooth regions) with an explicit method of tracking the location of discontinuity is employed. The goal of shock-capturing methods is to automatically capture discontinuities in the solution, without having to explicitly track them (Davis 1992).

## Review of numerical methods in solving hyperbolic PDEs

In solving the hyperbolic equations, traditional finite difference methods generate either non-physical oscillations or numerical diffusion in the presence of shocks (LeVeque 2004). This large error in the solution technique was the motivation behind the development of shock-capturing schemes. In this section, we review some of the most important techniques in solving the first-order hyperbolic problems and apply them in a numerical experiment to investigate the capability of each method.

### First-order finite difference schemes

First- or second-order finite difference methods have been traditionally used in capturing shocks. A very important family of the first-order schemes is the upwind methods, and the most popular upwind method is the Godunov scheme (Fennema and Chaudhry 1987). The direction of propagation of information (or waves) in this method is consistent with the spatial derivative discretization. Upwind schemes can cause strong diffusion and significant smearing in the solutions. In addition, the numerical method becomes very complex for nonlinear problems.

The Godunov scheme leads to finding the solution of a problem called the Riemann problem. An exact or approximate Riemann solver is required to solve the Riemann problem. An exact solver requires a high computational cost (Godunov 1959). Therefore, most numerical methods use approximate solvers (Engquist and Osher 1981; Harten et al. 1983; Roe 1981). The characteristics of the Jacobian matrix of the system construct the solution to the Riemann problem. Among approximate solvers, the Osher scheme (Engquist and Osher 1981) uses the signs of the eigenvalues to find the direction of the flux. On the other hand, the Roe scheme (Steger and Warming 1981) (flux difference splitting scheme) uses an average of the state variables calculated from either side of the Riemann interfacial values and approximates the Jacobian matrix. There are other approximate Riemann solvers such as Harten et al. (1983) and HHLC (Steger and Warming 1981). The details of these methods can be found elsewhere. In this paper, we have used the Godunov scheme in calculating the flux.

Besides the upwind methods, the Lax method is another popular first-order scheme. The centered difference discretization of the advection equation is unconditionally unstable. In the Lax or Lax–Friedrichs scheme (Lax 1954), the central difference scheme is stabilized by replacing the $Q_{i}^{n}$ term with the average $0.5\left( {Q_{i + 1}^{n} + Q_{i - 1}^{n} } \right)$ term in the discretization. The Lax method is known for its large dissipation error when the Courant number is not 1 and produces a leading phase error (Pletcher et al. 2012).

### Higher-order finite difference schemes

In most cases, first-order schemes are not employed to solve PDEs due to their intrinsic inaccuracy. Higher-order shock-capturing techniques are utilized to obtain better accuracy. The Lax–Wendroff scheme (Lax and Wendroff 1960), which is one of the earliest second-order finite difference schemes, can be obtained from the Taylor series expansion. The Lax–Wendroff scheme has predominantly lagging phase error except for large wave numbers with $0.5 < \nu < 1$, where $\nu$ is the Courant number. It is second-order accurate in both space and time.

There is another version of the Lax–Wendroff scheme, which is called the Richtmyer two-step Lax–Wendroff. This scheme is more suitable for nonlinear problems. It is second-order accurate with the same amplification factor and relative phase shift error as the original Lax–Wendroff. In the first step of this scheme, a Lax–Friedrichs scheme is applied at the midpoint for the half time step. For the remainder of the time step, a leap-frog scheme is applied. The Lax–Wendroff and two-step Lax–Wendroff schemes are equivalent when applied to linear advection equations.

The MacCormack method (Wesseling 2001) is a modified form of the two-step Lax–Wendroff scheme in which a temporary value of $Q_{i}^{n + 1}$ is calculated in the first step and is corrected in the second step. In the predictor equation, a forward difference for the space derivative is employed, while in the corrector equation a backward difference is used. The differencing scheme can be reversed, depending on the problem at hand.

The Beam–Warming scheme (Beam and Warming 1978) is a variation of the MacCormack method, which uses the same differencing in the predictor and corrector steps, depending on the sign of the velocity. This scheme, which is a second-order upwind scheme, has a predominantly leading phase error for $0 < \nu < 1$ and predominantly lagging phase error for $1 < \nu < 2$. On the other hand, the Lax–Wendroff method has opposite phase errors for $0 < \nu < 1$. Therefore, a linear combination of the two methods can reduce the dispersive error of the scheme. Fromm’s method of zero-average phase error (Wesseling 2001) is based on this observation.

There are a small number of third-order methods in the literature. Rusanov (1970) and Burstein and Mirin (1970), Warming et al. (1973) or tuned methods are among the famous schemes (Wesseling 2001) which we do not describe here.

High-order finite difference schemes are non-dissipative with good accuracy near the smooth regions. However, they are prone to generating spurious oscillations across discontinuities or in the vicinity of large gradients in the solution (LeVeque 2004). If the numerical oscillation becomes large, then the numerical methods become inefficient capturing an accurate solution.

### Artificial viscosity

**Fig. 1:**
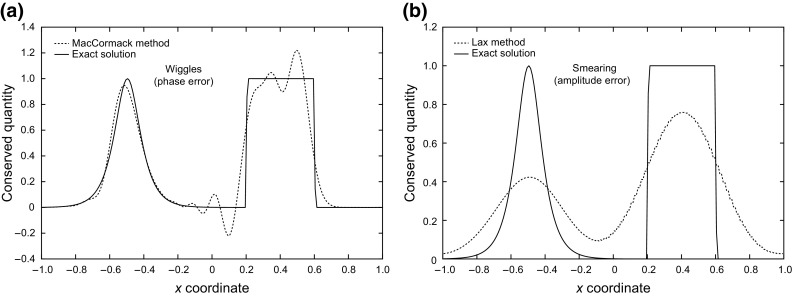
**a** Comparison of the MacCormack method result with the exact solution showing numerical distortion. **b** Comparison of the Lax method result with the exact solution showing numerical distortion dissipation

The Lax–Friedrichs, Lax–Wendroff and MacCormack methods belong to a class of solution methods that use artificial viscosity. This property is employed to introduce enough dissipation near discontinuities to smear oscillations (Fig. 1). The amount of this artificial viscosity should be negligible close to smooth regions (Fennema and Chaudhry 1990).

However, the difficulty with this approach is that it is hard to determine the amount of dissipation needed without causing unnecessary smearing. For this reason, the high-resolution methods were developed.

### High-resolution methods

In the past decade, attempts have been made to devise a method that can combine the monotone feature of first-order methods with the high accuracy of higher-order methods. This was achieved through high-resolution methods which are at least second-order accurate in smooth regions and non-oscillatory at discontinuities (LeVeque 2004). A measure of the oscillation is the total variation which is given by:

10 $${{TV}}\left( {Q^{n} } \right) = \mathop \sum \limits_{i = - \infty }^{ + \infty } \left| {Q_{i}^{n} - Q_{i - 1}^{n} } \right|$$

where *TV* is total variation.

It is obvious that in this definition more oscillations will give rise to more total variations. Therefore, to avoid oscillations it is necessary that the total variation decreases with time. Any numerical scheme that has this capability is called a total variation diminishing (TVD) scheme. In flux limiter schemes, limiters are imposed on the numerical flux function such that higher-order schemes are used in smooth regions, while lower-order schemes are employed close to the discontinuity. This combination can be achieved through:

11 $$F_{{i + \frac{1}{2}}}^{n} = F_{\text{L}} \left( {Q_{i} ,Q_{i + 1} } \right) + \phi_{{i + \frac{1}{2}}}^{n} \left[ {F_{\text{H}} \left( {Q_{i} ,Q_{i + 1} } \right) - F_{\text{L}} \left( {Q_{i} ,Q_{i + 1} } \right)} \right]$$

where $F_{\text{L}}$ denotes the lower-order flux function and $F_{\text{H}}$ denotes the higher-order flux function; $\phi_{{i + \frac{1}{2}}}^{n}$ is called the flux limiter which will be near zero closer to the discontinuities and around 1 close to the smooth data.

The definition of the limiter leads to a wide variety of other methods of this form. The flux-corrected transport (FCT) scheme of Boris and Book (1973) is one of the earliest limiter applications. Other popular choices of limiters include the superbee limiter (Roe 1985), van Leer limiter (van Leer 1977), Woodward limiter (Colella 1985), Minmod limiter (Colella 1985) and the monotone upstream-centered scheme for conservation laws (MUSCL) by van Leer (Colella 1985).

### Dimensional splitting

Dimensional splitting is a type of fractional step method which can be used to transform a PDE with a source term to a homogeneous, ordinary differential equation. The two subproblems can be solved independently, with any scheme for each subproblem. It can also be applied in converting a multi-dimensional problem into several one-dimensional problems. The advection equation in 2-D with a source term can be written as:

12 $$\frac{\partial q}{\partial t} + u\left( {x,y} \right)\frac{\partial q}{\partial x} + v\left( {x,y} \right)\frac{\partial q}{\partial y} = \psi \left( x \right)$$

where $u$ and $v$ are velocities in the $x$ and $y$ directions and $\psi \left( x \right)$ is a source term. In proppant injection applications, the source term is the injection rate of proppants. Now, applying dimensional splitting to Eq. (12), we obtain:

13 $$\frac{\partial q}{\partial t} = \psi \left( x \right)$$

14 $$\frac{\partial q}{\partial t} + u\left( {x,y} \right)\frac{\partial q}{\partial x} = 0$$

15 $$\frac{\partial q}{\partial t} + v\left( {x,y} \right)\frac{\partial q}{\partial y} = 0$$

Equation (13) is an ordinary differential equation (ODE) and can be integrated using standard methods for solving ODEs (e.g., Euler or Runge–Kutta methods). In the next step, the high-resolution methods can be applied to Eqs. (14) and (15) without change to advect the solution.

This method can also be used to discretize the fracture along the width direction without generating elements with high aspect ratios.

### Benchmark test to evaluate different solution techniques

To test the capability of the capturing techniques discussed here, we consider a 1-D linear advection equation [Eq. (3)] with unit velocity (Garcia-Navarro and Vazquez-Cendon 2000).

16 $$\frac{\partial q}{\partial t} + \frac{\partial q}{\partial x} = 0,\quad - 1 < x < 1$$

For the initial condition of our numerical experiment, we consider a combination of a Gaussian wave, a square wave, a sharp triangle and a half ellipse:

17 $$q\left( {x,0} \right) = q_{0} \left( x \right)$$

18 $$q_{0} (x) = \left\{ {\begin{array}{*{20}l} {\frac{1}{6}(G(x,\beta ,z - \delta ) + G(x,\beta ,z + \delta ) + 4G(x,\beta ,z)),} \hfill & { - 0.8 \le x \le - 0.6} \hfill \\ {1,} \hfill & { - 0.4 \le x \le - 0.2} \hfill \\ {1 - \left| {10\left( {x - 0.1} \right)} \right|,} \hfill & {0 \le x \le 0.2} \hfill \\ {\frac{1}{6}(H(x,\alpha ,a - \delta ) + H(x,\alpha ,a + \delta ) + 4H(x,\alpha ,a)),} \hfill & { 0.4 \le x \le 0.6} \hfill \\ {0,} \hfill & {\text{otherwise}} \hfill \\ \end{array} } \right.$$

The functions *G* and *H* are defined as:

19 $$G\left( {x,\beta ,z} \right) = {\text{e}}^{{ - \beta \left( {x - z} \right)^{2} }}$$

20 $$H\left( {x,\alpha ,a} \right) = \sqrt {\hbox{max} \left( {1 - \alpha^{2} \left( {x - a} \right)^{2} } \right),0}$$

Also we set:

21 $$z = - 0.7, \quad \delta = 0.005,\quad \beta = \frac{\log \left( 2 \right)}{{36\delta^{2} }},\quad a = 0.5,\quad \alpha = 10$$

The exact solution of this problem is the translation of the initial solution at unit speed:

22 $$q\left( {x,t} \right) = q_{0} \left( {x - t} \right)$$

**Fig. 2:**
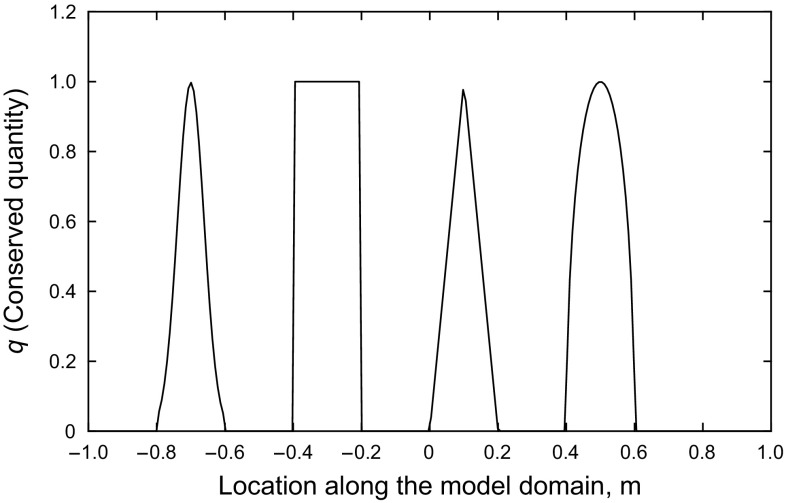
Numerical experiment with Gaussian, square, sharp triangle, and half ellipse initial waves

Figure 2 shows a schematic of the initial condition.

We used a periodic boundary condition on the left and right sides of the model and ran the simulation for 2 s. For this model problem, we assigned a Courant number of 0.9 for all the schemes.

**Fig. 3:**
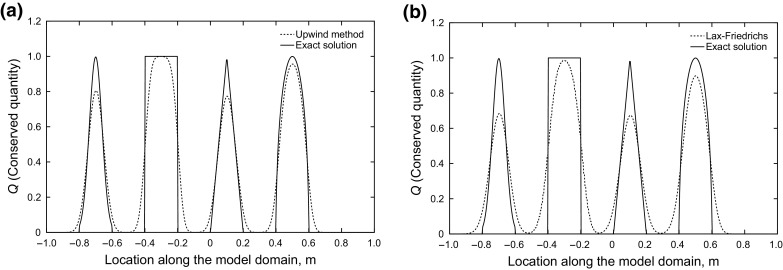
Results of first-order schemes after 2 s, upwind (left) and Lax–Friedrichs (right), monotonic without oscillation

Figure 3 shows that the first-order upwind and Lax–Friedrichs methods are monotone everywhere. In other words, they do not lead to oscillations anywhere in the solution. However, they have poor accuracy due to large dissipation.

**Fig. 4:**
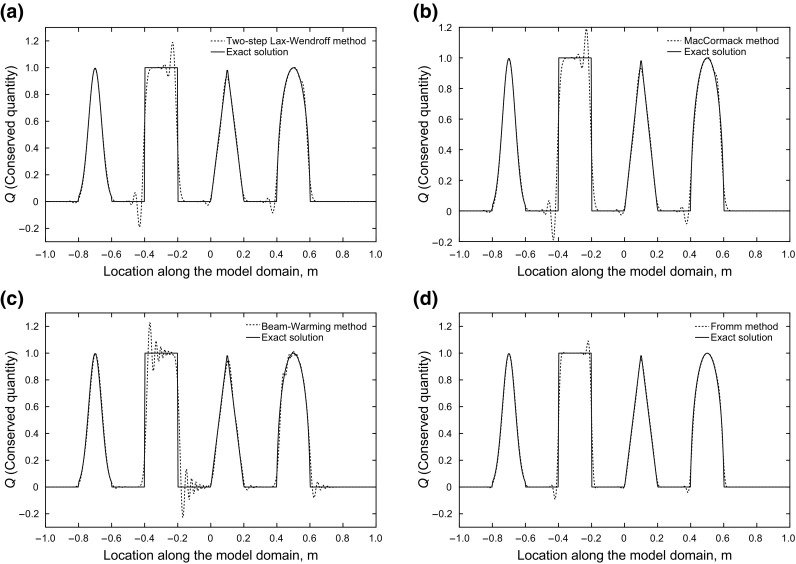
Results of second-order schemes after 2 s, accurate in smooth regions, oscillatory near shocks

On the other hand, Fig. 4 shows that the high-order methods provide good accuracy in smooth regions, while giving oscillations close to discontinuities. The oscillations happening in the vicinity of the discontinuity show the dispersive nature of these methods.

**Fig. 5:**
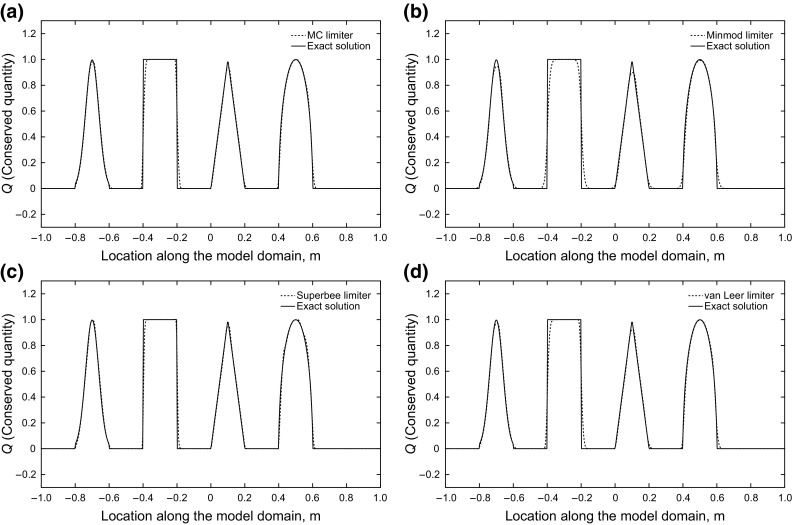
Flux limiter schemes: accurate in smooth regions and near shocks

Figure 5 presents the results of the simulations with high-resolution methods. It is obvious that the first-order schemes show no oscillation and good accuracy is obtained in the higher-order schemes.

## Application of the proposed technique to proppant transport problems

To provide a numerical solution for the proppant transport partial differential equation, we applied a simple iteration approach (Ertekin et al. 2001). The partial differential equation that we obtained in the previous section is nonlinear, meaning that the coefficients of the equation depend on the unknown. It is true for the proppant mass balance equation. At each time step, the transport problem is divided into three parts:

1. First, we calculate the coefficients of the slurry mass balance equation at the previous time step (at time level *n*) or at the previous iteration level, *k*. We solve for the pressure field using the viscosity, density and width obtained from the previous time step (or the initial conditions for the first time step).
2. Next, we calculate the velocity of the proppant, using the pressure field calculated in the previous time step.
3. Finally, we apply a finite volume method to advect the concentration of proppant.

**Fig. 6:**
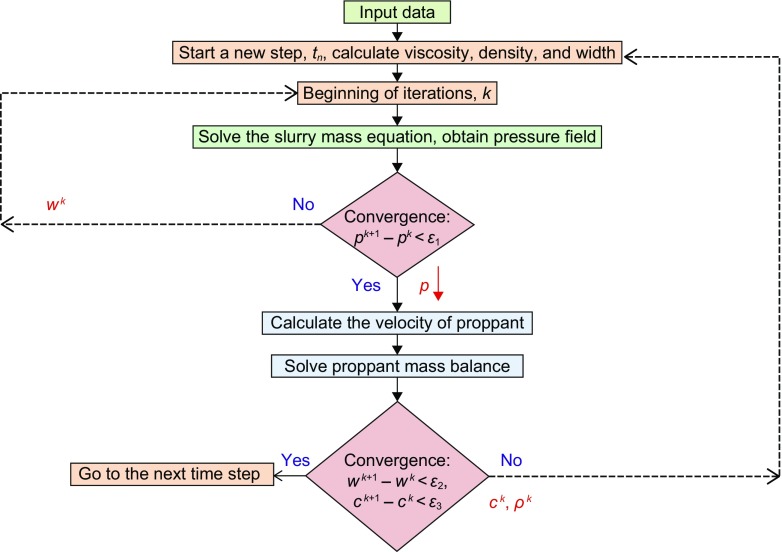
Numerical algorithm of solving system of mass balance equations

We iterate on the solution until convergence is achieved. Figure 6 shows the coupling between the slurry and proppant mass balance solvers.

### Proppant and slurry mass balance (continuity equation)

A continuity equation is basically a statement based on the conservation of mass. In the proppant transport context, two mass conservation equations are required for the slurry which consists of proppant and injection fluid. The derivation of these balance equations can be found elsewhere (Barree and Conway 1995), and here, we only mention these conservation equations.

For the proppant:

23 $$\frac{\partial }{\partial t}\left( {\rho_{\text{p}} cw} \right) + \frac{\partial }{\partial x}\left( {\rho_{\text{p}} u_{\text{p}} cw} \right) + \frac{\partial }{\partial y}\left( {\rho_{\text{p}} v_{\text{p}} cw} \right) + cq_{\text{inj}} = 0$$

For the injection fluid:

24 $$\frac{\partial }{\partial t}\left( {\rho_{\text{f}} \left( {1 - c} \right)w} \right) + \frac{\partial }{\partial x}\left( {\rho_{\text{f}} u_{\text{f}} \left( {1 - c} \right)w} \right) + \frac{\partial }{\partial y}\left( {\rho_{\text{f}} v_{\text{f}} \left( {1 - c} \right)w} \right) + \left( {1 - c} \right)q_{\text{inj}} = 0$$

where $w$ is the fracture width; $c$ is the volumetric proppant concentration defined as the ratio of proppant volume to slurry volume; $u_{\text{p}}$ and $u_{\text{f}}$ are horizontal velocities of proppant and fluid; $v_{\text{p}}$ and $v_{\text{f}}$ are vertical velocities of proppant and fluid; $\rho_{\text{p}}$ and $\rho_{\text{f}}$ are proppant and fluid densities; and $q_{\text{inj}}$ is the slurry injection flow rate.

For the binary system of proppant flow, Bird et al. (1960) applied Fick’s law of mass diffusion and included a diffusion term in the above mass balance equation of the form:

25 $$J = \nabla \left( {\rho wD_{\text{pf}} \nabla \omega_{\text{p}} } \right)$$

where $J$ is the diffusion flux, $\rho$ is the slurry density; $D_{\text{pf}}$ is the diffusivity coefficient; and $\omega_{\text{p}}$ is the mass fraction of the proppant which is defined as:

26 $$\omega_{\text{p}} = \frac{{c\rho_{\text{p}} }}{\rho }$$

The diffusivity can be broken into the following three terms:

27 $$D_{\text{pf}} = D_{\text{pf}}^{\text{mo}} + D_{\text{pf}}^{\text{tu}} + D_{\text{pf}}^{\text{te}}$$

The term $D_{\text{pf}}^{\text{mo}}$ is the diffusion associated with the molecular movement, $D_{\text{pf}}^{\text{tu}}$ with the turbulent flow and $D_{\text{pf}}^{\text{te}}$ with the temperature gradient. The diffusion associated with molecular movement is usually very small in the slurry injection problem. Also, assumptions are made that the flow of frac fluid is laminar and that the entire field is in a constant temperature field. For these reasons, the diffusive term has been dropped from the mass balance equation.

If we eliminate the density terms in Eqs. (23) and (24) and add them together, we can obtain the mass balance equation for the slurry:

28 $$\frac{\partial w}{\partial t} + \frac{\partial }{\partial x}\left( {u_{\text{sl}} w} \right) + \frac{\partial }{\partial y}\left( {v_{\text{sl}} w} \right) + q_{\text{inj}} = 0$$

where $u_{\text{sl}}$ and $v_{\text{sl}}$ are the horizontal and vertical velocities of the slurry defined as:

29 $$u_{\text{sl}} = cu_{\text{p}} + \left( {1 - c} \right)u_{\text{f}}$$

30 $$v_{\text{sl}} = cv_{\text{p}} + \left( {1 - c} \right)v_{\text{f}}.$$

### Conservation form versus advection form for proppant transport

There are several forms that a variable-coefficient hyperbolic partial differential equation might take, each of which arises depending on the context. The numerical solution technique may depend on the form of the PDE. The hyperbolic proppant transport equation [Eq. (1)] is written in the advection form by extending the space derivatives:

31 $$\frac{\partial }{\partial t}\left( {\rho_{\text{p}} cw} \right) + \rho_{\text{p}} u_{\text{p}} \frac{\partial }{\partial x}\left( {cw} \right) + cw\frac{\partial }{\partial x}\left( {\rho_{\text{p}} u_{\text{p}} } \right) + \rho_{\text{p}} v_{\text{p}} \frac{\partial }{\partial y}\left( {cw} \right) + cw\frac{\partial }{\partial y}\left( {\rho_{\text{p}} v_{\text{p}} } \right) + cq_{\text{inj}} = 0$$

Since the proppant density is assumed to be constant under the flow conditions, by eliminating $\rho_{\text{p}}$ we obtain:

32 $$\frac{\partial }{\partial t}\left( {cw} \right) + u_{\text{p}} \frac{\partial }{\partial x}\left( {cw} \right) + cw\frac{\partial }{\partial x}\left( {u_{\text{p}} } \right) + v_{\text{p}} \frac{\partial }{\partial y}\left( {cw} \right) + cw\frac{\partial }{\partial y}\left( {v_{\text{p}} } \right) + cq_{\text{inj}} = 0$$

By factoring out the term $cw$, a familiar form of continuity equation appears:

33 $$\frac{\partial }{\partial t}\left( {cw} \right) + u_{\text{p}} \frac{\partial }{\partial x}\left( {cw} \right) + v_{\text{p}} \frac{\partial }{\partial y}\left( {cw} \right) + cw\left[ {\frac{\partial }{\partial x}\left( {u_{\text{p}} } \right) + \frac{\partial }{\partial y}\left( {v_{\text{p}} } \right)} \right] + cq_{\text{inj}} = 0$$

However, we know that for an incompressible fluid, due to the continuity equation the term in the brackets will be zero. Therefore, the advection form of the proppant transport equation will be:

34 $$\frac{\partial }{\partial t}\left( {cw} \right) + u_{\text{p}} \frac{\partial }{\partial x}\left( {cw} \right) + v_{\text{p}} \frac{\partial }{\partial y}\left( {cw} \right) + cq_{\text{inj}} = 0$$

This form of equation will require a simpler form of high-resolution methods.

### Analytical solution of the Navier–Stokes equation: cubic law

The Navier–Stokes equation describes the motion of a fluid as a relationship between flow velocity (or momentum) and pressure. It is also called the momentum conservation equation. The momentum conservation law states that the forces acting on a small element of fluid accelerate that element. This equation is equivalent to Newton’s second law of motion.

In hydraulic fracturing applications, an analytical solution to the Navier–Stokes equation is used for the fluid flow inside the fractures. This solution is called the cubic law (Economides and Nolte 2000):

35 $$u_{\text{sl}} = - \frac{{w^{2} }}{12\mu }\frac{\partial p}{\partial x}$$

Similarly, for the *y* direction:

36 $$v_{\text{sl}} = - \frac{{w^{2} }}{12\mu }\frac{{\partial \left( {p - \rho gy} \right)}}{\partial y}$$

where $p$ is pressure; $g$ is acceleration due to gravity; and $\mu$ is viscosity.

Now, if we substitute Eqs. (35) and (36) into the slurry mass balance, Eq. (28), we obtain:

37 $$\frac{\partial }{\partial x}\left( {\frac{{w^{3} }}{12\mu }\frac{\partial p}{\partial x}} \right) + \frac{\partial }{\partial y}\left( {\frac{{w^{3} }}{12\mu }\frac{{\partial \left( {p - \rho gy} \right)}}{\partial y}} \right) + q_{\text{inj}} = \frac{\partial w}{\partial t}$$

In this equation, the viscosity and density depend on the concentration and the fracture width is also non-constant. Therefore, we are dealing with a nonlinear partial differential equation and we need a suitable technique to linearize our PDE.

## Simulation example

Our transport model consists of coupling mass balance of slurry and proppant. The slurry mass balance equation is an elliptic PDE and is solved by an implicit method, and the shock-capturing methods discussed earlier are not applied to solve this PDE. We applied different shock-capturing techniques to the 2-D proppant transport problem in a fixed slot to examine their performance with respect to accuracy and spurious oscillations. We developed our 2-D models through the application of dimensional splitting. Finally, we presented the result of proppant injection into the fixed slot using the best-performing numerical technique until the whole fracture was filled up with proppants.

We applied Godunov dimensional splitting as explained in Sect. 3.5 to this 2-D problem. It is also noted that the temporal term in Eq. (34) will become zero for this problem since the width of the opening is constant.

### Model specifications

**Fig. 7:**
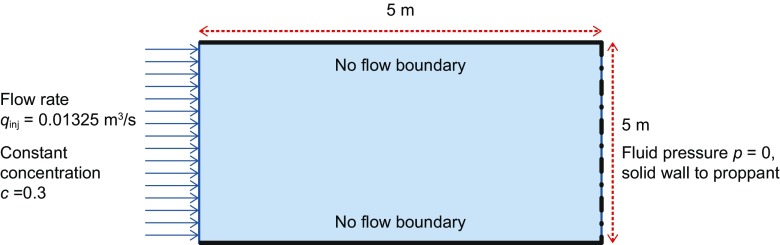
Model domain and applied boundary conditions to slurry and proppant transport equations

The model domain is a 5 m by 5 m by 0.0061 m slot. Figure 7 shows the domain and applied boundary conditions. The slurry injection was assigned a rate of 0.01325 m^3^/s with 0.3 (dimensionless) proppant concentration at the left boundary (inlet). Top, bottom and right boundaries were assigned zero proppant flux. Fluid was assigned zero pressure at the right boundary but was not allowed to exit from the top and bottom boundaries.

In proppant transport problems, the concentration can vary between 0 and a value that is called saturation concentration. The saturation concentration corresponds to the maximum solid concentration in a slurry. For regular spheres, the saturation concentration corresponds theoretically to a random packing. For real material, it can be determined experimentally and depends on the type of the proppant and varies between 0.52 (loose packing) and 0.65 (dense packing). We assigned a value of 0.6 to this parameter in the simulations. As the solid loading increases, the viscosity of the slurry changes. We assumed the increase in viscosity follows a trend according to (Barree and Conway 1994):

38 $$\mu = \frac{{\mu_{0} }}{{\left( {1 - \frac{c}{{c^{*} }}} \right)^{1.82} }}$$

where $c^{*}$ is the saturation concentration and $\mu_{0}$ is the initial viscosity of the clean fluid that was water in our case with a density of 1000 kg/m^3^.

Also it was assumed that particles settle in accordance with the corrected Stokes equation (Stokes 1850) as proposed by Govier and Aziz (1972) (mentioned in Barree and Conway 1994):

39 $$V_{\text{set}} = \frac{{g\left( {\rho_{\text{p}} - \rho_{\text{f}} } \right)d_{\text{p}}^{2} }}{18\mu }{\text{e}}^{ - 5.9c}$$

where $V_{\text{set}}$ is the settling velocity of proppant particles and $d_{\text{p}}$ is the proppant diameter.

### Mesh sensitivity

More accurate results can be obtained by using finer mesh in the finite difference scheme. However, the solution and computational time may become excessively large. Therefore, there is a trade-off between the accuracy of a refined model and the running time of the analysis and data processing time. The purpose of mesh sensitivity analysis in this section is to find an adequate, yet reasonable, mesh size capable of giving good accuracy in a reasonable time.

**Fig. 8:**
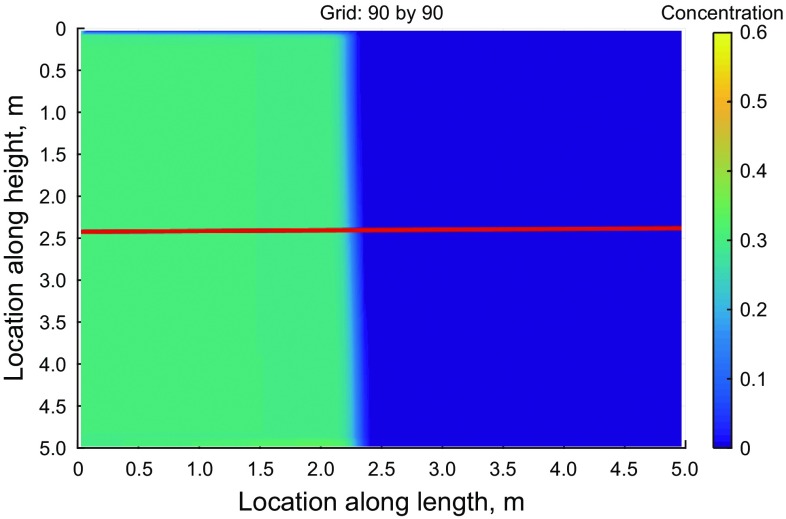
Proppant concentration after 5 s of injection

**Fig. 9:**
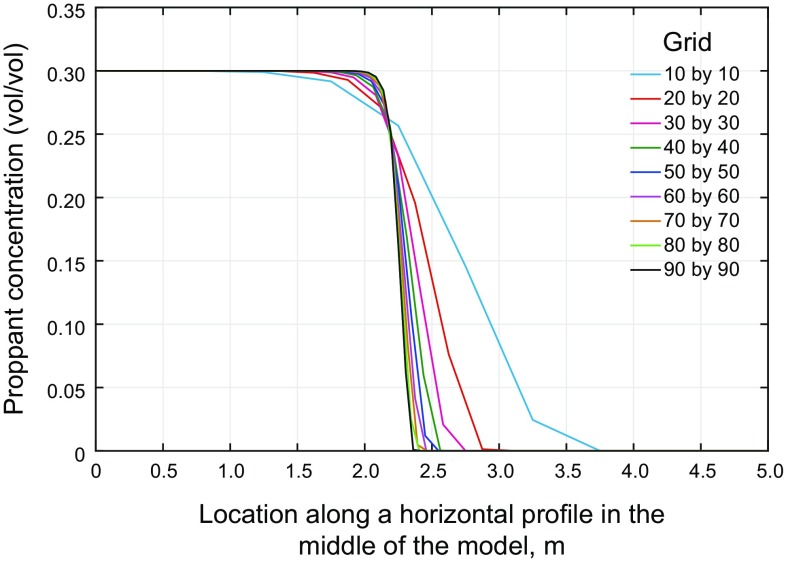
Mesh sensitivity results of concentration along the horizontal profile in the middle of the model

For all the simulations of this part, the superbee flux limiter was utilized. The number of the elements in the $x$ and $y$ directions was varied from 10 by 10 grids to 90 by 90, in increments of 10. Figure 8 shows the concentration map after 5 s of injection for the finest mesh. As shown in the figure, we chose a cross section exactly in the middle of the domain and plotted the concentration for different grid sizes in Fig. 9.

Figure 9 shows that the resulting concentration curve converges by reducing the mesh size. Since the difference in the results between 60 by 60 and 90 by 90 grids is small, we chose 60 by 60 grids for the rest of the simulations.

### Simulation results

In this section, we present the result using the first-order upwind, second-order Lax–Wendroff, superbee and van Leer flux limiters method.

**Fig. 10:**
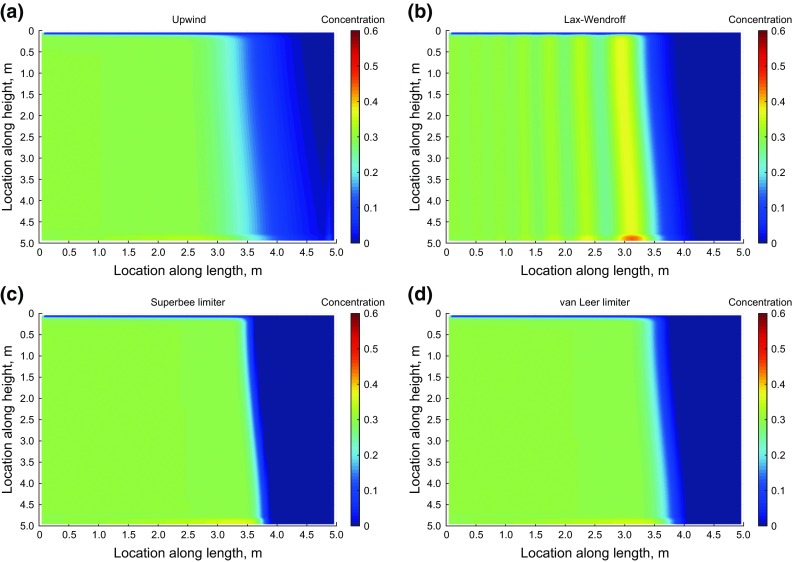
Application of **a** upwind, **b** Lax–Wendroff, **c** superbee and **d** van Leer flux limiters to proppant transport

Figure 10 shows the proppant distribution in the model after 8 s of injection. The oscillatory behavior of the high-order Lax–Wendroff is apparent from the figure. The upwind method in this figure shows a gradual decrease in concentration at the front. Such behavior can happen only if some diffusion exists in the original PDE, while, as we discussed earlier in Sect. 4.1, we have neglected the diffusion in the proppant balance equation. Therefore, smearing of the result and poor capability of the solution scheme in capturing the shock leads to this behavior. On the other hand, the superbee and van Leer flux limiters not only give non-oscillatory results, but also capture the shock front with no smearing.

**Fig. 11:**
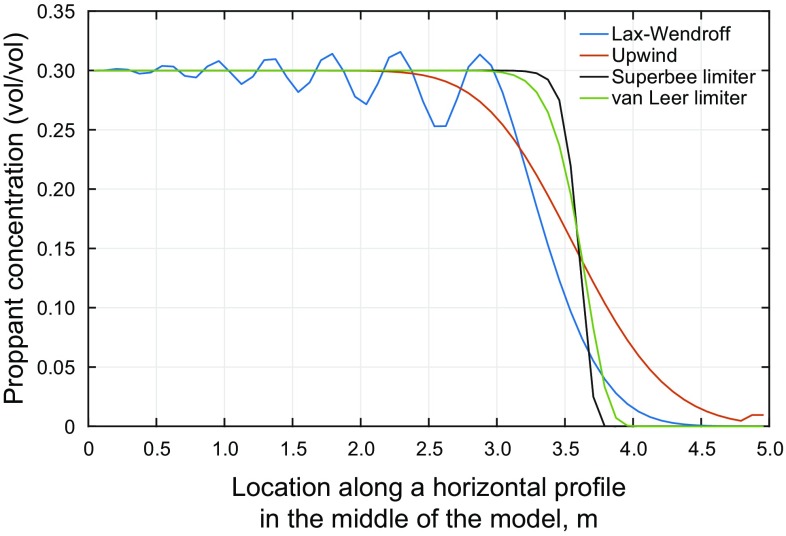
Comparison of different solution techniques for the concentration along the horizontal profile in the middle of the model (see Fig. 10)

The oscillatory behavior of Lax–Wendroff and inaccuracy of the upwind method is more obvious in Fig. 11. In this figure, we again plotted the concentration profile along the horizontal section at the middle of the model. The superbee limiter method is effectively capturing the shock front, while the upwind and van Leer Limiter methods are smearing the results near the shock front. The Lax–Wendroff method also results in spurious oscillations, which is not desirable.

**Fig. 12:**
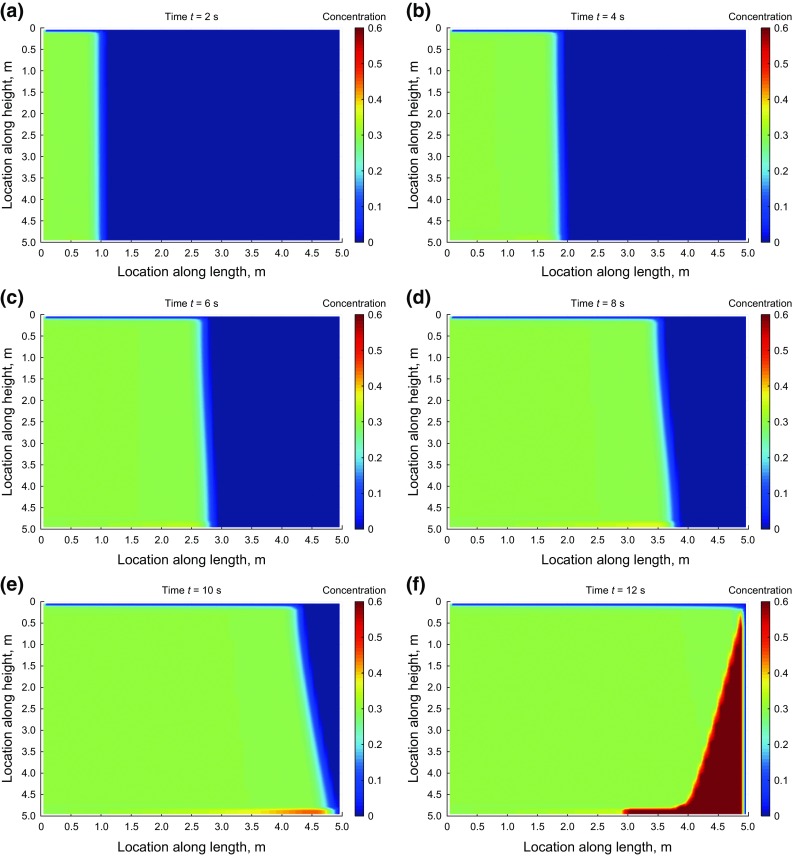
Concentration profiles during injection at 2–12 s

**Fig. 13:**
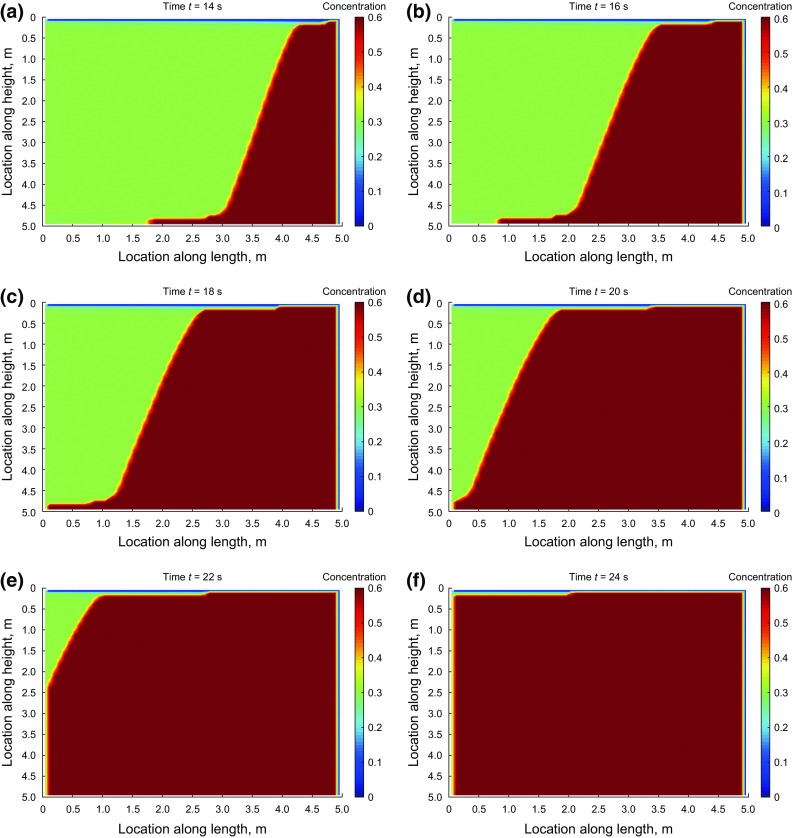
Concentration profiles during injection at 14–24 s

Figures 12 and 13 show the concentration maps at different times during the injection using the superbee flux limiter as the numerical technique. We continued the simulation until the whole model was filled up with proppant. The input data and model boundary conditions are the same as previously described.

Due to much higher horizontal velocity of proppant relative to its vertical velocity in the specified condition of the simulation, little settlement is observed at the bottom of the slot. However, a proppant bank is created at the discharge part of the model. The bank grows with time in a slightly asymmetrical manner due to the presence of a small component of proppant vertical velocity.

## Conclusions

1. First-order finite difference schemes are always monotonic preserving. However, they are not accurate enough near smooth regions of solution.
2. Although higher-order finite difference schemes give good accuracy in smooth regions, they produce spurious oscillations near regions with high gradients in the solution.
3. High-resolution finite volume methods through the application of flux limiters can be employed in simulating proppant transport since they are always non-oscillatory (total variation diminishing) near the location with large gradients. These schemes also produce accurate results in the smooth regions.
4. The Godunov splitting technique is very effective in simulating multi-dimensional problems. Applying this method eliminates unnecessary complexity of the un-split methods and makes modeling easier for coding.
